# Editorial: Neuromodulatory Interventions for Pain

**DOI:** 10.3389/fnins.2021.746328

**Published:** 2021-08-13

**Authors:** Trevor Thompson, Lauren C. Heathcote, Hannah Hobson, Marco Solmi

**Affiliations:** ^1^Centre for Chronic Illness and Ageing, University of Greenwich, London, United Kingdom; ^2^Department of Anesthesiology, Perioperative, and Pain Medicine, Stanford University School of Medicine, Stanford, CA, United States; ^3^Department of Psychology, University of York, York, United Kingdom; ^4^Department of Neuroscience, University of Padova, Padua, Italy

**Keywords:** neuromodulation, pain, neurofeedback, acupuncture, non-invasive brain stimulation, TMS, tDCS

The opioid crisis has prompted a renewed interest in non-pharmacological interventions for pain, with recent promising evidence for their effectiveness (Pillai Riddell et al., 2015; Veehof et al., 2016; Thompson et al., 2017, 2019; Franco et al., 2018; Owen et al., 2020). So called neuromodulatory interventions, which include neurofeedback and electrical and magnetic brain stimulation, are a novel class of non-pharmacological treatments that have attracted both intrigue and controversy. Despite a growing research literature, there remains a sense of uncertainty over whether they represent realistic alternatives to pain medication.

The aim of this research topic was to stimulate new, much-needed research evaluating the effectiveness of such treatments and providing insights into their possible mechanisms. We present a collection of 11 articles focused primarily on neurofeedback, non-invasive brain stimulation and acupuncture that attempt to address some of these issues.

## Neurofeedback

A number of studies examined neurofeedback (NFB). This involves providing users with visual or auditory feedback on their brain activity (usually from EEG or fMRI), with the aim of assisting them in self-regulating this activity in a way that will produce favorable effects on pain.

A systematic review of 24 studies by Roy et al. provided a comprehensive summary of the current evidence for NFB across a range of pain conditions. The authors concluded that most studies identified improvements in pain, fatigue, sleep, and mood, but that heterogeneity in study protocols made it impossible to determine an optimal protocol for NFB administration. There was some evidence that regulation of EEG/MRI activity was possible, but this was not consistent across studies. Interestingly, the authors noted that improvement in pain sometimes occurred whether or not the targeted brain activity was successfully changed, suggesting a likely non-specific therapeutic component to NFB. They also found an encouraging improvement in study quality over the last few years, which included the increased use of control groups. Several limitations were nevertheless noted, with infrequent use of sham groups or randomization.

Some of these limitations were tackled in a new primary study by Terrasa et al., which randomized 17 fibromyalgia patients to 6 sessions of sensorimotor rhythm (SMR) training (*n* = 9) or a sham false-feedback procedure (*n* = 8). They found that 4 patients in the SMR training group who showed some ability to modulate SMR (“good” responders) also showed significant reductions in pain, with no such changes observed for “bad” responders or controls. Ide-Walters and Thompson randomized 24 healthy participants to receive 10 x NFB (with real EEG feedback) or 10 × sham (with false EEG feedback) sessions and assessed their responses to experimentally-induced cold pain. NFB was based on individualized protocols determined by initial qEEG assessments. While a significant decrease in pain across sessions was found for the NFB group, a near identical decrease was found for the sham group, consistent with the idea that any therapeutic effects of NFB could be at least in part attributable to a non-specific component. Vučković et al. examined the feasibility of self-administering NFB as a home treatment. After initial training in alpha upregulation and theta downregulation, 15 chronic pain patients with spinal cord injuries were asked to practice sessions at home for several weeks. Twelve patients showed statistically significant reductions in pain, with 8 showing clinically significant (>30%) reductions. Training was particularly successful when an individualized alpha target frequency was used, based on the participant's dominant alpha peak frequency (7.6 Hz) rather than a fixed frequency band (8–12 Hz). Such results suggest NFB could be feasible for self-administration, providing appropriate training and oversight are given.

An interesting alternative approach to alpha regulation was explored by Arendsen et al. They attempted to directly manipulate alpha activity in 20 chronic musculoskeletal pain patients using a novel visual stimulation procedure. Although no significant impact on pain was found, global alpha power was significantly higher during 10 Hz (alpha) stimulation than other frequencies. This suggests visual stimulation may be effective at regulating alpha activity, and this method could therefore warrant further investigation.

## Non-Invasive Brain Stimulation

Non-invasive brain and spinal stimulation methods have also been used to treat chronic pain. Zucchella et al. conducted a systematic review of studies assessing the effectiveness of these techniques for pain in individuals with multiple sclerosis. They reviewed 9 studies, that included direct current (tDCS) and magnetic stimulation (TMS) applied primarily to the left dorsolateral pre-frontal cortex or primary motor cortex. Consistent improvements in pain were identified, although there was less evidence for beneficial effects for broader well-being symptoms such as depression, fatigue, cognition, and quality of life. These findings suggest that the promising results that have been previously found for brain stimulation in conditions such as neuropathy and fibromyalgia may extend to pain in multiple sclerosis, although the authors caution that better controlled studies that assess longer term outcomes are still needed. A novel case study by Nguyen et al. provided preliminary evidence that the potential benefits of rTMS might also apply to knee osteoarthritis. Stimulation of the motor cortex in a 71-year-old woman who exhibited evidence of central sensitization of pain was linked to substantial improvements in pain, sleep and fatigue and these were still evident at the end of treatment almost 1 year after treatment commenced.

## Acupuncture and Cognitive Therapy

Several knockout mice studies also examined potential mechanisms that might explain how central nervous system activity linked to pain processing is modulated by interventions such as acupuncture. Zhu et al. used an induced pain paradigm in mice and found a reduction in pain hypersensitivity following electroacupuncture. They also found evidence for bidirectional regulation of GABAergic neurons and glutamatergic neurons via the CB1 receptors in the ventrolateral periaqueductal gray as a likely key analgesic mechanism. Jang et al. also found that acupuncture reduced pain behavior in mice, and identified an elevation in the expression levels of glutamate receptors in the hippocampus as the likely mechanism. The importance of the modulation of glutamine activity in altering pain processing was corroborated by a review of the two clinically approved glutamate modulators acetyl-L-carnitine and ketamine by Freo et al.

In a different approach, Timmers et al. examined how neuromodulatory changes resulting from a form of cognitive-behavioral therapy to reduce pain-related fear may be beneficial for patients with low back pain. fMRI showed that the changes that occurred in the right posterior insula and medial prefrontal cortex in patients (but not controls) during presentation of pain-related imagery did not occur after treatment. Other observed patterns led the authors to conclude that the neural circuitry for pain-related fear was modulated by the therapy, and that these changes were associated with decreases in pain-related fear.

Our collection brings together a diverse set of research papers on the issue of pain and potential neuromodulatory interventions for its management.

## Author Contributions

TT wrote the manuscript. LH, HH, and MS contributed to the revision, read, and approved the submitted version. All authors contributed to the article and approved the submitted version.

## Conflict of Interest

The authors declare that the research was conducted in the absence of any commercial or financial relationships that could be construed as a potential conflict of interest.

## Publisher's Note

All claims expressed in this article are solely those of the authors and do not necessarily represent those of their affiliated organizations, or those of the publisher, the editors and the reviewers. Any product that may be evaluated in this article, or claim that may be made by its manufacturer, is not guaranteed or endorsed by the publisher.
